# Supplementary material for: Structural landscape of the respiratory syncytial virus nucleocapsids
Source: Nat Commun. 2023 Sep 15;14:5732. doi: 10.1038/s41467-023-41439-8 (PMC10504348; doi:10.1038/s41467-023-41439-8)
Supplement: Supplementary file 1 — Supplementary Information File [file 41467_2023_41439_MOESM1_ESM.pdf]

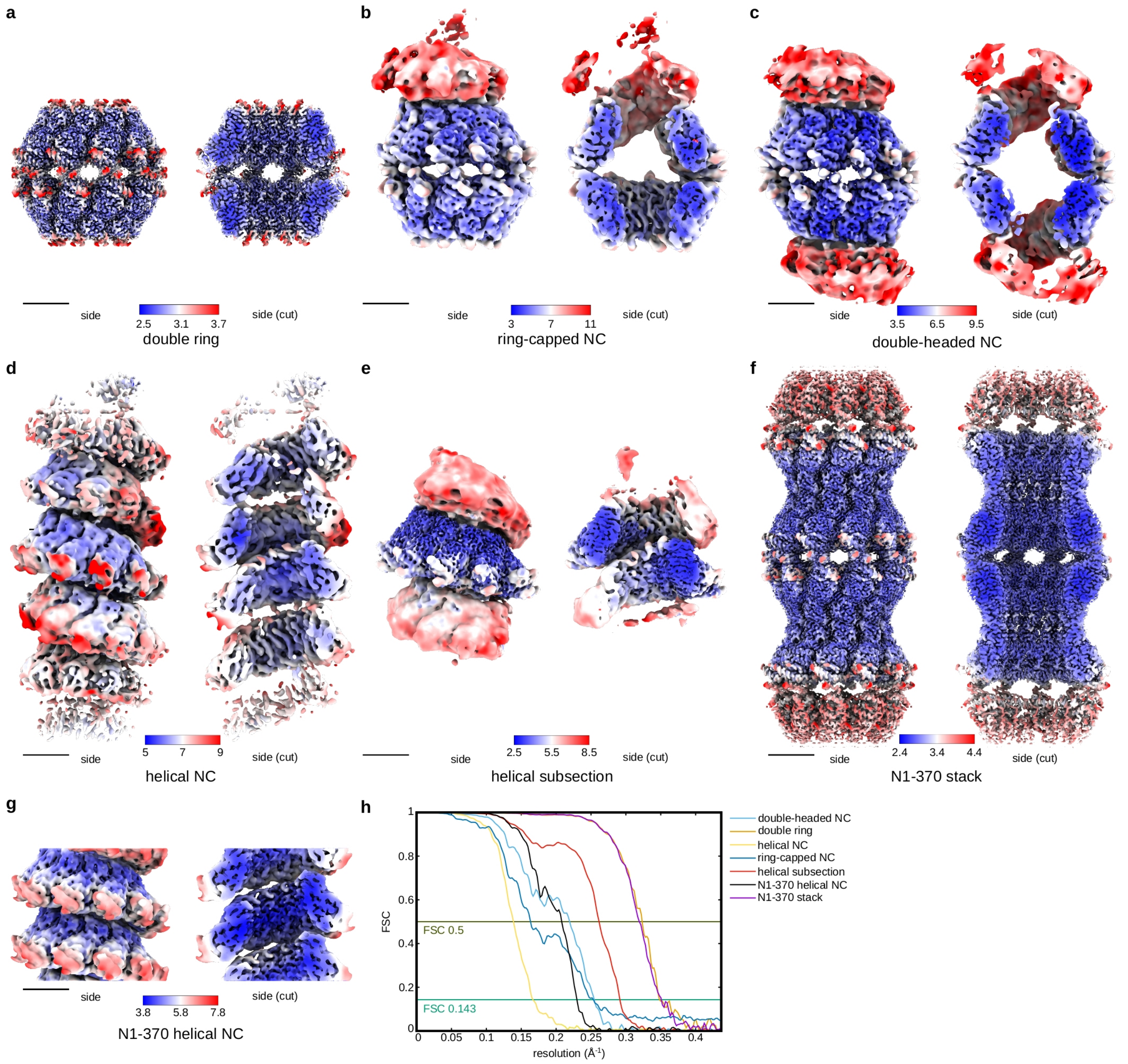

Supplementary Figure 1. Cryo-EM maps determined in this work and the corresponding FSC curves. Cryo-EM maps filtered and coloured by local resolution (in Å) for the double ring (a), ring-capped NC (b), double-headed NC (c), helical NC (d), helical subsection (e), N1-370 stack (f) and N1-370 helical NC (g). Contours levels used in ChimeraX to generate the full or cut-out views are 0.264 (a), 0.096 (b), 0.103 (c), 0.117 (d), 0.084 (e), 0.174 (f) and 0.075 (g). (h) Masked FSC curves. Scale bars 50 Å.

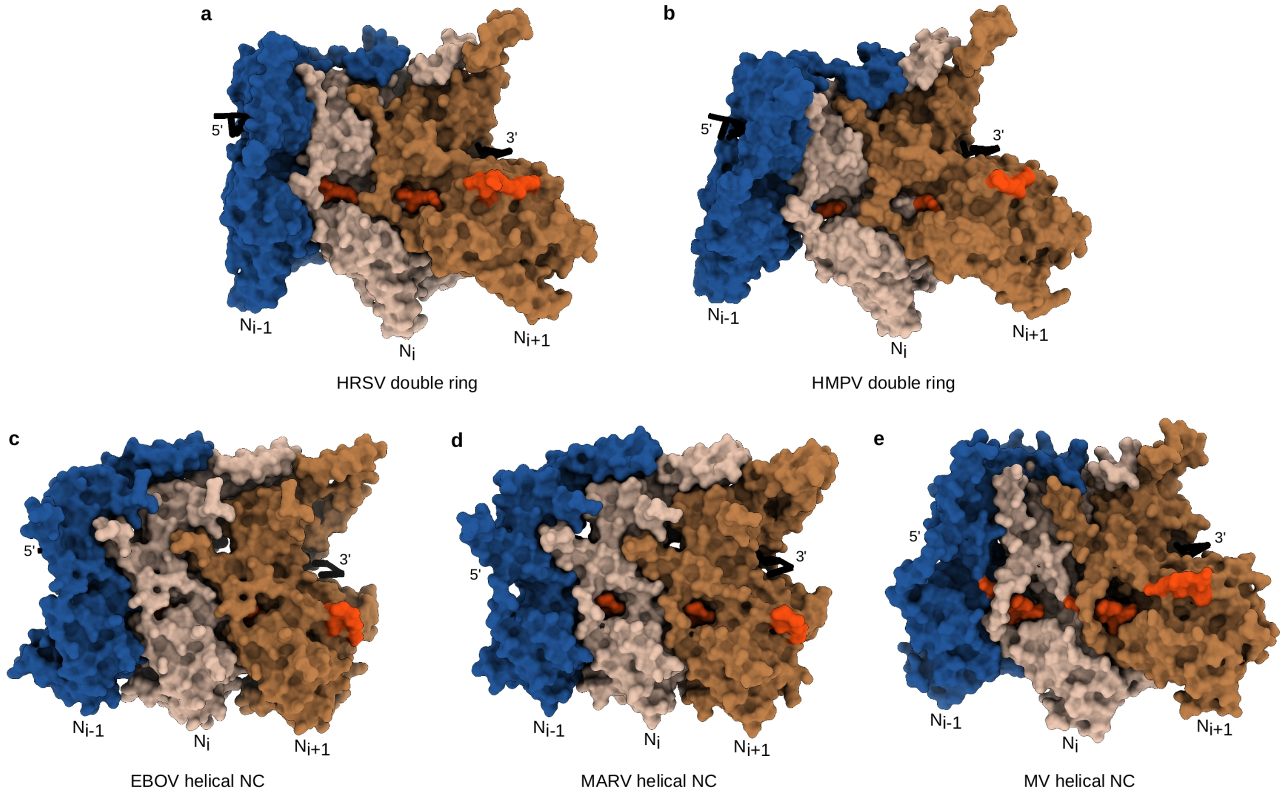

Supplementary Figure 2. N-hole conservation between *Pneumoviridae*, *Filoviridae* and *Paramyxoviridae* families. Atomic models of three consecutive protomers of the cryo-EM structure of HRSV double ring (a), crystal structure of the HMPV double ring (PDB: 5FVC) (b), Ebola virus helical NC (PDB: 5Z9W) (c), Marburg virus helical NC (PDB: 7F1M) (d), and Measles virus helical NC (PDB: 4UFT) (e) are shown as a surface. The residues coloured in orange correspond to the short loop from the NTD of the N_{i-1} protruding into the N-hole of N_i, numbered 230-238 in HRSV N (a), 234-238 in HMPV N (b), 221-226 in EBOV N (c), 203-208 in MARV N (d) and 242-248 in MV (e). Of note, EBOV N-hole is less visible because the short loop (in orange) protruding into the N-hole is masked by the loops forming the N-hole. RNA is in black.

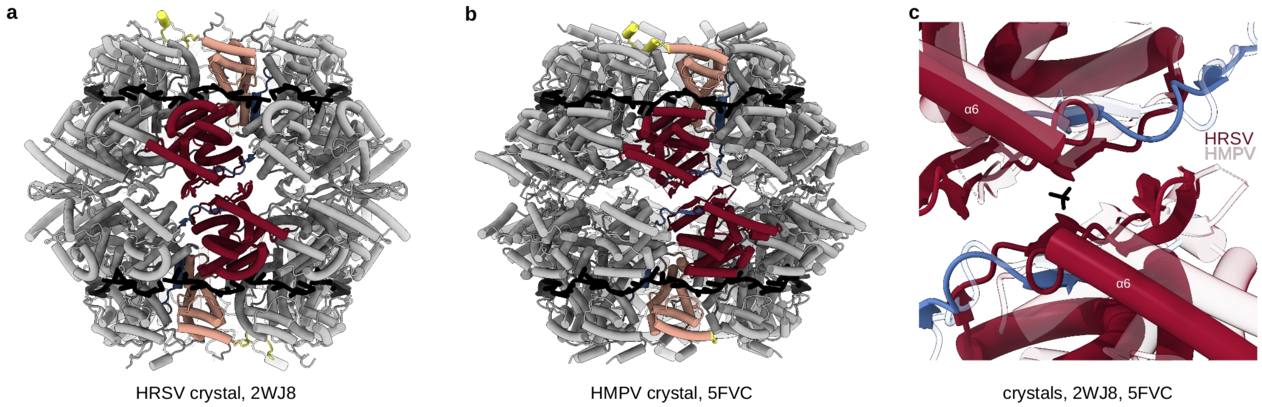

Supplementary Figure 3. Comparison of HRSV and HMPV double rings as observed in the respective crystals. (a) Atomic model of the HRSV N_{10} double ring (PDB: 2WJ8) is shown as cartoon, coloured as in Figure 1. (b) Atomic model of the HMPV N_{10} double ring (PDB: 5FVC). (c) Close-up of the alignment of the HRSV and HMPV N_{10} double rings. HRSV N_{10} double ring is coloured as in (a) and HMPV N_{10} double ring is in transparent. The borate ion in the interaction site in PDB: 2WJ8 is shown. The 6th alpha-helix is labeled for reference.

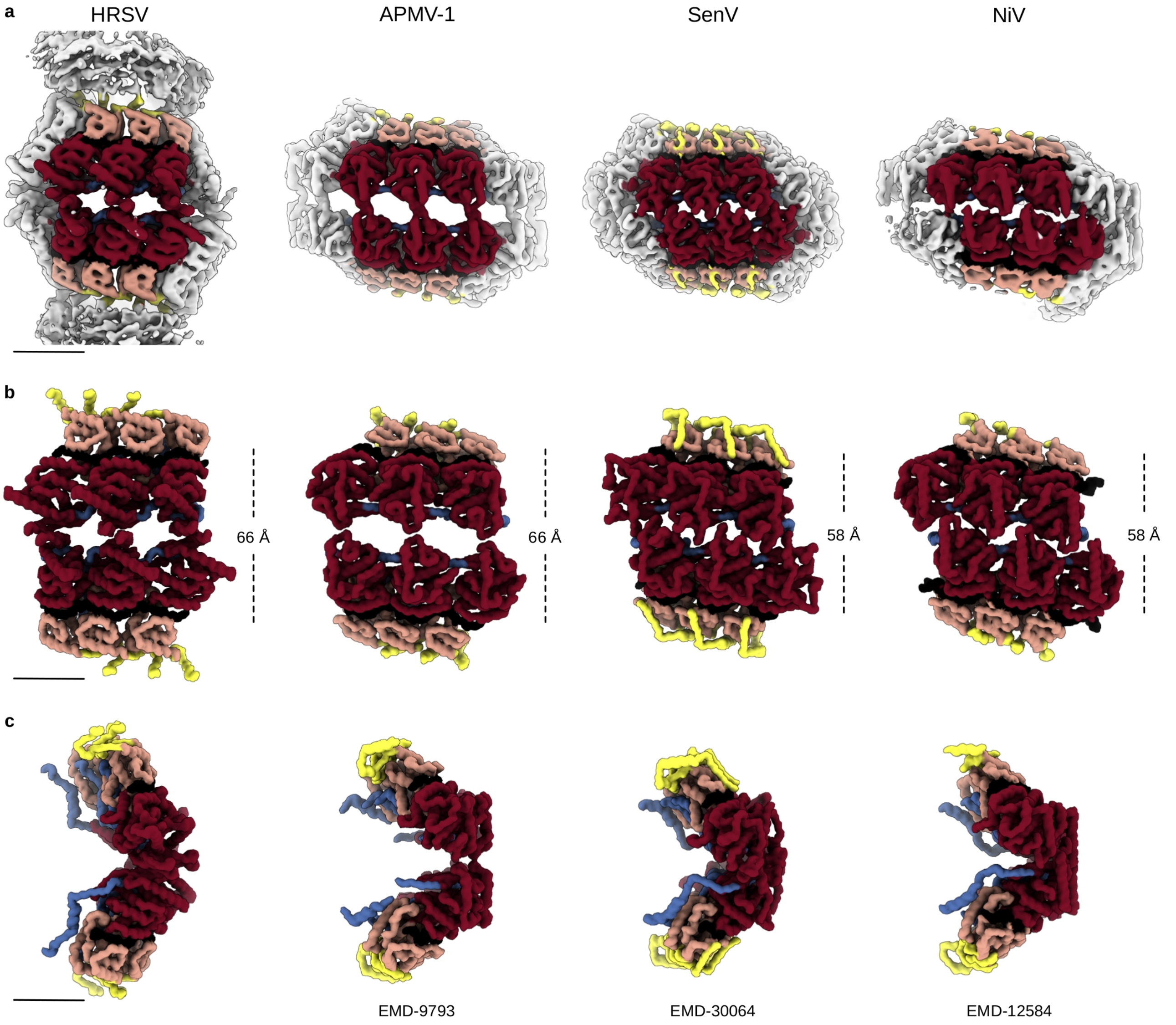

Supplementary Figure 4. Comparison of double-headed NCs of HRSV, APMV-1, SenV and NiV. (a) Cryo-EM maps of double-headed NCs of HRSV, Newcastle disease or avian paramyxovirus 1 (APMV-1) (EMD-9793), Sendai virus (SenV) (EMD-30064) and Nipah virus (NiV) (EMD-12584), filtered to 6 Å resolution, scale bar 50 Å. Front (b) and side (c) views of atomic models of three consecutive protomers from the double-headed NCs of HRSV, APMV-1, SenV and NiV, filtered to 6 Å resolution and displayed as surface. Of note, the clam-shaped assemblies of HRSV and AMPV-1 are both formed in an end-on fashion, without insertion into the opposite inter-protomer grooves and with the centers of gravity of the two spirals separated by 66 Å, as in the cryo-EM structure of the HRSV N_{10} double ring. In contrast, the SenV and NiV clams adopt a closely nested packing, leading to inter-spiral distances of only 58 and 56 Å respectively, and therefore actually look more reminiscent of the crystal structure of the HRSV double ring. A visual comparison of the side views of the central clam-forming protomers, opposite to the helical junction, also reveals that the the HRSV protomer is notably less tilted from the filament axis than its three paramyxoviral counterparts.

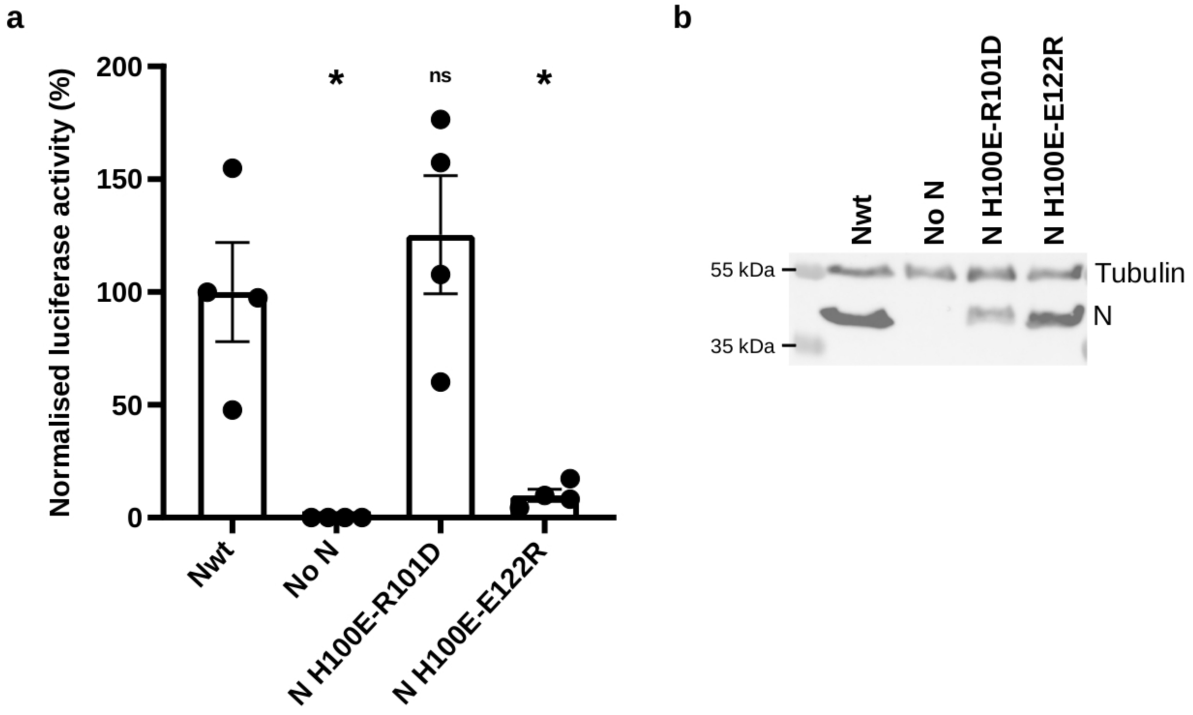

Supplementary Figure 5. Impact of N mutation on the HRSV polymerase activity. (a) Quantification of the HRSV polymerase activity in the presence of wild type N (Nwt), in the absence of N (No N) and for the two N mutants, H100E-R101D and H100E-E122R. Luciferase activities were normalised based on corresponding ß-Gal activities, and are representative of three experiments performed in quadruplicates. Data are mean +/- s.e.m. (standard error of the mean). n = 4 replicates. A two-tailed Mann-Whitney test was used to compare the normalised luciferase activities between the Nwt and the other conditions. $p < 0.05$ is considered significant. *, p = 0.0286. For N H100E-R101D, p = 0.3429 (ns for no significance). (b) Western blot showing the expression of N (WT and mutants) and tubulin in BSRT7/5 cells. Source data are provided as a Source Data file.

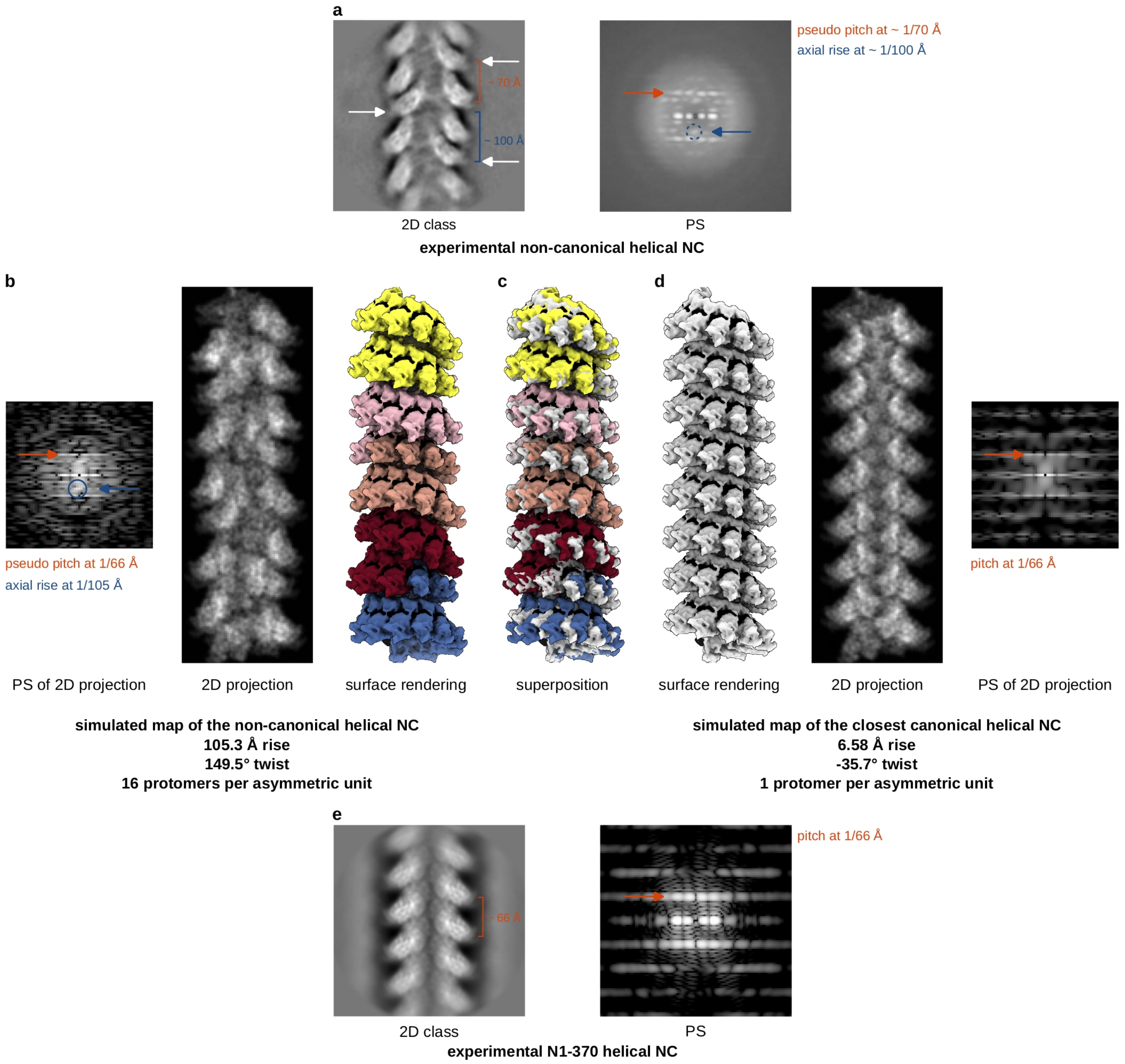

Supplementary Figure 6. Comparison between the experimental NC, the simulated non-canonical helical NC, the simulated closest canonical helical NC and the N1-370 mutant NC. (a) A 2D class-average (left) and an average of the PS of the particles in the class (right), representative of the experimental non-canonical helical NCs are shown. The white arrows are indicating the inwards-shifted protomers. (b) A simulated non-canonical helical NC map has been built using the experimentally refined helical parameters as indicated and filtered to 8 Å resolution (each asymmetric unit is coloured in a different colour, right). The corresponding 2D projection (middle) and its PS (left) are shown. (c) Superposition of the simulated maps of the non-canonical helical NC and the closest canonical helical NC. (d) The closest canonical helical NC symmetry parameters were calculated by dividing the non-canonical helical rise and twist by the number of protomers in the asymmetric unit (16; 105.3/16=6.58 Å rise and a -(360-149.5+360)/16=-35.7° twist to account for right-handed to left-handed conversion). The corresponding simulated map filtered to 8 Å resolution (left), its 2D projection (middle) and its PS (right) are shown. (e) A 2D class-average (left) and an average of the PS of the particles in the class (right), representative of the canonical helical NC formed by the N1-370 mutant construct.

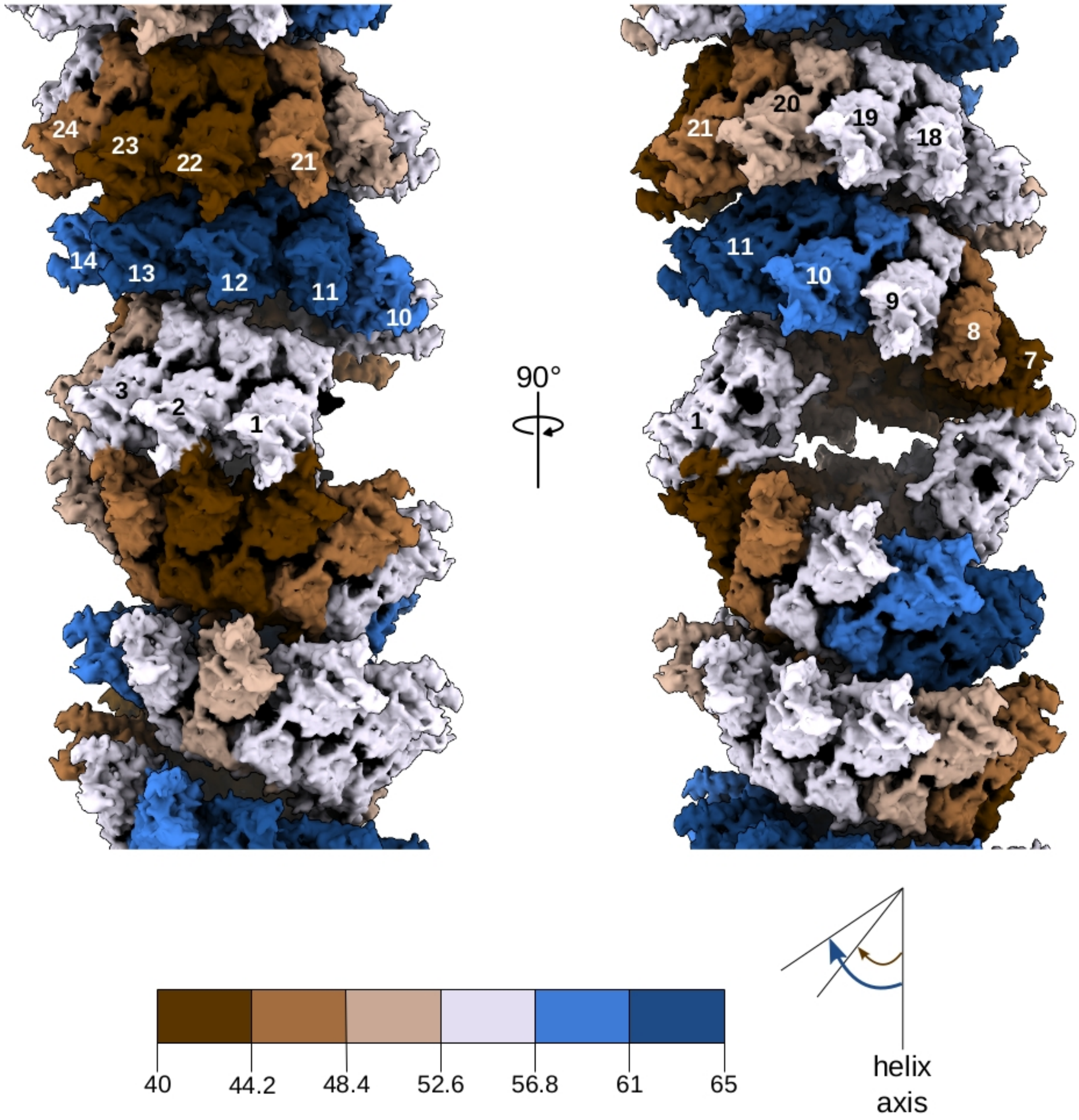

Supplementary Figure 7. Model of a long double-headed NC. A simulated map of long double-headed NCs, filtered to 6 Å resolution, was built from the atomic model of the double-headed NC. Protomers are coloured dependent on their axial tilt determined as described in the method section, following the colour code shown at the schematic underneath, RNA is in black. The protomer numbering follows the convention used for the non-canonical helical NC.

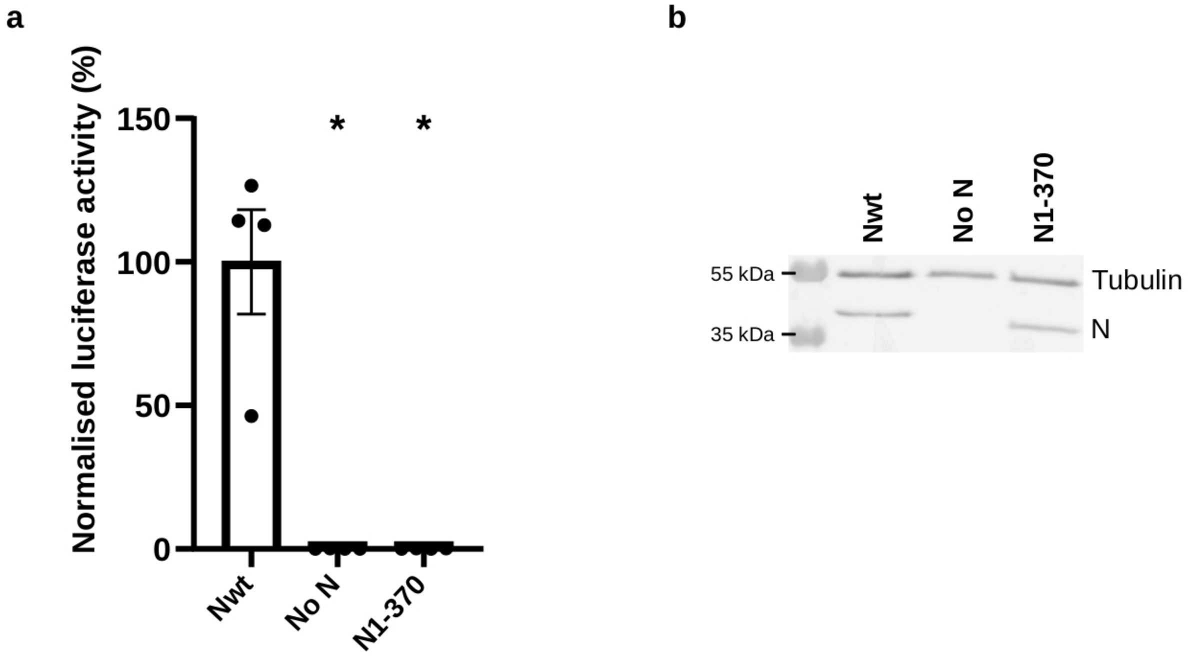

Supplementary Figure 8. Impact of the deletion of CTD-arm of N on the HRSV polymerase activity. (a) Quantification of the HRSV polymerase activity in the presence of wild type N (Nwt) or N1-370, or in the absence of N (No N). Luciferase activities were normalised based on corresponding ß-Gal activities, and are representative of two experiments performed in quadruplicates. Data are mean +/- s.e.m. (standard error of the mean). n = 4 replicates. A two-tailed Mann-Whitney test was used to compare the normalised luciferase activities between the Nwt and the other conditions. $p < 0.05$ is considered significant. *, $p = 0.0286$. (b) Western blot showing the expression of N (WT and mutant) and tubulin in BSRT7/5 cells. Source data are provided as a Source Data file.

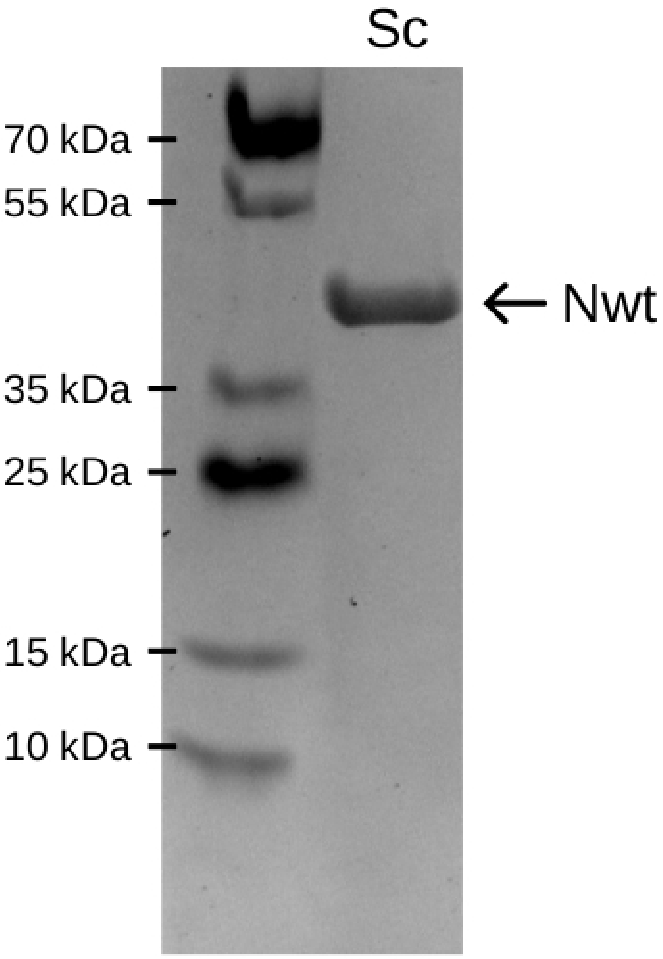

Supplementary Figure 9. Purification of NC sample for cryo-EM. The NC preparation used for cryo-EM was analysed by SDS-PAGE coloured with Coomassie blue. Sc: supernatant after concentration, Nwt: wild type N. The gel shows the absence of the C-terminal peptide of HRSV P in the final sample. Source data is provided as a Source Data file.

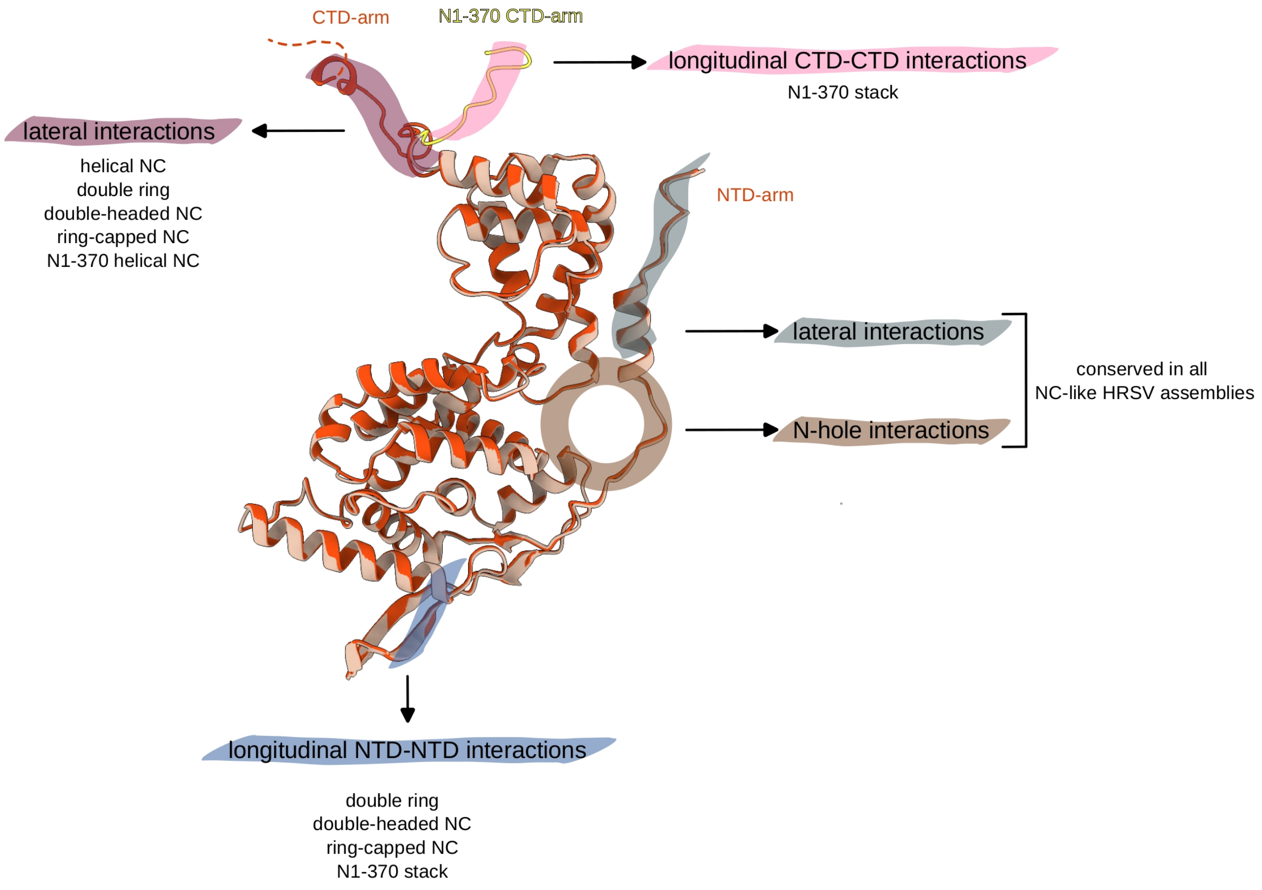

Supplementary Figure 10. Schematics of involvement of specific structural elements on N in interaction interfaces formed upon assembly of different types of HRSV NC-like particles. Alignement of N protomers of the HRSV N10 double ring (orange) and the N1-370 stack (beige). Positions of the NTD- and CTD-arms are indicated.

Supplementary Table 1. Primer sequences used to generate pN deletion and mutations

Plasmid name	Primer sequence (5’-3’)
pN1-370	Forward ACTAGACTTGtaaGCAGAAGAACTAG<br>Reverse ACACTGTAGTTAATCACAC
pNH100E	Forward TGTAACAACAgagCGTCAAGACAT<br>Reverse CTACTCCATTTGCTTTTAC
pNH100E-R101D	Forward AACAACAGAGgacCAAGACAT<br>Reverse ACATCTACTCCATTTGCTTTTAC
pNH100E-E122R	Forward CTTAACAACTgacATTCAAATCAAC<br>Reverse CTTGCCAATGTTAACACTTC

The pNH100E plasmid was used as a template to generate the plasmids pNH100E-R101D and pNH100E-E122R.

Supplementary Table 2. Cryo-EM data collection, refinement and validation statistics

**Dataset**	**WT RSV NC**					**Mutant 1-370 RSV NC**	
**Name of the maps generated in this study**	**N10 double ring**	**Helical NC**	**Ring-capped NC**	**Double-headed NC**	**Helical sub-section**	**Helical NC**	**Stack**
**EMDB PDB**	EMD: 17031 PDB: 8OOU	EMD: 17030	EMD: 17037	EMD: 17036	EMD: 17035 PDB: 8OP1	EMD: 17034	EMD: 17038 PDB: 8OP2
**Data collection and processing**							
Magnification			36,000			36,000	
Voltage (kV)			200			200	
Electron exposure (e–/Å^2)			42.0			42.0	
Defocus range (µm)			-0.7 – -2.4			-0.7 – -2.4	
Pixel size (Å)			1.145			1.145	
Symmetry imposed	D10	Helical 149.5° 105.3Å	C1	D1	C1	Helical -36° 6.58Å	D10
Initial particle images (no.)	-	1406835	-	-	-	471,549	-
Final particle images (no.)	47,212	389,540	22,162	25,338	389,540	329,706	81,918
Map resolution (Å) FSC threshold	2.9 0.143	6.2 0.143	3.9 0.143	3.8 0.143	3.5 0.143	4.3 0.143	2.8 0.143
Map resolution range (Å)	2.5-3.7	5-9	3-11	3.5-9.5	2.5-8.5	3.8-7.8	2.4-4.4
**Refinement**							
Initial model used (PDB code)	2WJ8	-	-	-	2WJ8	-	2WJ8
Model resolution (Å) (masked) FSC threshold	2.9 0.5	-	-	-	3.6 0.5	-	2.98 0.5
Map sharpening *B* factor (Å^2)	-96 Å^{-2}	-	-	-	-94 Å^{-2}	-	-99 Å^{-2}
Model composition Non-hydrogen atoms Protein residues Ligands	61,800 7,560 0	-	-	-	15,415 1890 0	-	120,560 14,760 0
B factors (Å^{-2}) Protein	38.96	-	-	-	43.37	-	47.85
R.m.s. deviations Bond lengths (Å) Bond angles (°)	0.005 0.628	-	-	-	0.006 0.739	-	0.005 0.883
Validation MolProbity score Clashscore Poor rotamers (%)	1.50 8.30 0.00	-	-	-	2.03 12.61 0.00	-	2.18 8.64 5.19
Ramachandran plot Favoured (%) Allowed (%) Outliers (%)	97.77 2.23 0.00	-	-	-	93.67 6.33 0.00	-	97.00 3.00 0.00
