# Supplementary material for: Structural landscape of the respiratory syncytial virus nucleocapsids
Source: Nat Commun. 2023 Sep 15;14:5732. doi: 10.1038/s41467-023-41439-8 (PMC10504348; doi:10.1038/s41467-023-41439-8)
Supplement: Supplementary file 3 — Description of Additional Supplementary Files [file 41467_2023_41439_MOESM3_ESM.pdf]

Description of Additional Supplementary Files

Supplementary Movie 1 : Morphing of the non-canonical full-length helical NC to the canonical N1-370 helical NC. Differences between the non-canonical full-length helical NC and the canonical N1-370 helical NC structures as viewed from the side and from the top. Atomic model of the non-canonical helical NC is displayed as surface and coloured as in Figure 5. Atomic model of the canonical N1-370 helical NC is displayed as surface and coloured in dark grey. RNA is in black.
