# Supplementary material for: Structural landscape of the respiratory syncytial virus nucleocapsids
Source: Nat Commun. 2023 Sep 15;14:5732. doi: 10.1038/s41467-023-41439-8 (PMC10504348; doi:10.1038/s41467-023-41439-8)
Supplement: Supplementary file 5 — Reporting Summary [file 41467_2023_41439_MOESM5_ESM.pdf]

nature portfolio

Corresponding author(s): Irina Gutsche
Ambroise Desfosses

Last updated by author(s): Aug 26, 2023

Reporting Summary

Nature Portfolio wishes to improve the reproducibility of the work that we publish. This form provides structure for consistency and transparency in reporting. For further information on Nature Portfolio policies, see our Editorial Policies and the Editorial Policy Checklist.

Statistics

For all statistical analyses, confirm that the following items are present in the figure legend, table legend, main text, or Methods section.

n/a	Confirmed	
☐	☒	The exact sample size (*n*) for each experimental group/condition, given as a discrete number and unit of measurement
☐	☒	A statement on whether measurements were taken from distinct samples or whether the same sample was measured repeatedly
☐	☒	The statistical test(s) used AND whether they are one- or two-sided<br>*Only common tests should be described solely by name; describe more complex techniques in the Methods section.*
☒	☐	A description of all covariates tested
☐	☒	A description of any assumptions or corrections, such as tests of normality and adjustment for multiple comparisons
☐	☒	A full description of the statistical parameters including central tendency (e.g. means) or other basic estimates (e.g. regression coefficient) AND variation (e.g. standard deviation) or associated estimates of uncertainty (e.g. confidence intervals)
☐	☒	For null hypothesis testing, the test statistic (e.g. *F*, *t*, *r*) with confidence intervals, effect sizes, degrees of freedom and *P* value noted<br>*Give P values as exact values whenever suitable.*
☒	☐	For Bayesian analysis, information on the choice of priors and Markov chain Monte Carlo settings
☒	☐	For hierarchical and complex designs, identification of the appropriate level for tests and full reporting of outcomes
☒	☐	Estimates of effect sizes (e.g. Cohen's *d*, Pearson's *r*), indicating how they were calculated

Our web collection on statistics for biologists contains articles on many of the points above.

Software and code

Policy information about availability of computer code

Data collection	Image Lab Touch Software version 2.3.0.07, Tecan i-control 2.0, SerialEM 4.0
Data analysis	Chimera 1.16 , ChimeraX 1.4, Coot 0.9.6.2, GraphPad Prism 9, Phenix 1.19.2-4158, RELION 4.0, cryoSPARC 3.3.2, EMAN2 2.99, CrYOLO 1.9.3, bsoft 2.1.3

For manuscripts utilizing custom algorithms or software that are central to the research but not yet described in published literature, software must be made available to editors and reviewers. We strongly encourage code deposition in a community repository (e.g. GitHub). See the Nature Portfolio guidelines for submitting code & software for further information.

Data

Policy information about availability of data

All manuscripts must include a data availability statement. This statement should provide the following information, where applicable:

- Accession codes, unique identifiers, or web links for publicly available datasets
- A description of any restrictions on data availability
- For clinical datasets or third party data, please ensure that the statement adheres to our policy

The coordinates and structure factors generated in this study (Supplementary Table 2) have been deposited in the EM Data Bank (EMDB) and Protein Data Bank (PDB) under accession codes EMD-17031, PDB: 8OOU (double-ring), EMD-17030 (non-canonical helical NC), EMD-17035, PDB: 8OP1 (helical subsection), EMD-17036 (double-headed NC), EMD-17037 (ring-capped NC), EMD-17034 (canonical helical NC formed by the N1-370 mutant), and EMD-17038, PDB: 8OP2 (stack formed by the N1-370 mutant). The data underlying Figure 4c and Supplementary Figures 5, 8 and 9 generated in this study are provided in the

Supplementary Information/Source Data file.

Human research participants

Policy information about studies involving human research participants and Sex and Gender in Research.

Reporting on sex and gender	N/A
Population characteristics	N/A
Recruitment	N/A
Ethics oversight	N/A

Note that full information on the approval of the study protocol must also be provided in the manuscript.

Field-specific reporting

Please select the one below that is the best fit for your research. If you are not sure, read the appropriate sections before making your selection.

☒ Life sciences ☐ Behavioural & social sciences ☐ Ecological, evolutionary & environmental sciences

For a reference copy of the document with all sections, see nature.com/documents/nr-reporting-summary-flat.pdf

Life sciences study design

All studies must disclose on these points even when the disclosure is negative.

Sample size	For the minigenome assay, sample size was determined based on previous studies using the same method and shown to be appropriate, see doi:10.1128/JVI.00058-12 or doi:10.1128/JVI.00909-21. The experiment was done three times, in quadruplicate. For cryo-EM analysis, the numbers of micrographs collected and particles used are indicated in the methods section and inSupplementary Table 2. The numbers of micrographs used correspond to one or two days session on a Thermofisher Glacios microscope operated with Serial EM. It was chosen to allow to determine the structures of RSV NCs at the resolution described in the manuscript.
Data exclusions	No data were excluded for the minigenome assay. The numbers of particles retained for the final cryo-EM reconstructions are indicated in the methods section.
Replication	Protein expression and purification was performed five times for the wild type and twice for the truncation mutant NCs and gave similar results. For the minigenome assay, the experiment was done three times, in quadruplicate and gave similar results. Data presented are from one representative experiment. Twelve cryo-EM grids were screened for the wild type and eight for the truncation mutant NCs, showing similar particle assemblies for characteristic for the respective NC type. Grids with the best ice quality were selected for data collection.
Randomization	N/A for the minigenome assay because it is a cell culture-based assay: each well contains cells transfected with various plasmids, and the wells must be identified along the experiment, from transfection to data collection, in order to attribute the results to the correct mix of plasmids. For cryo-EM analysis, refinement was carried out according to the gold-standard refinement protocol, which involves random assignment of particles to half-datasets.
Blinding	N/A because predetermined samples and conditions were used throughout the study.

Reporting for specific materials, systems and methods

We require information from authors about some types of materials, experimental systems and methods used in many studies. Here, indicate whether each material, system or method listed is relevant to your study. If you are not sure if a list item applies to your research, read the appropriate section before selecting a response.

Materials & experimental systems

n/a	Involved in the study	
☐	☒	Antibodies
☐	☒	Eukaryotic cell lines
☒	☐	Palaeontology and archaeology
☒	☐	Animals and other organisms
☒	☐	Clinical data
☒	☐	Dual use research of concern

Methods

n/a	Involved in the study	
☒	☐	ChIP-seq
☒	☐	Flow cytometry
☒	☐	MRI-based neuroimaging

Antibodies

Antibodies used	Rabbit polyclonal anti-N antibody (dilution 1/2000)<br>Mouse monoclonal anti-alpha-tubulin antibody (Sigma, product number T6199, clone DM1A, lot 115M4796V)<br>Anti-rabbit secondary antibody, peroxidase-labeled (SeraCare, material number 5450-0010, lot 10437708)<br>Anti-mouse secondary antibody, peroxidase-labeled (SeraCare, material number 5450-0011, lot 10430730)
Validation	Rabbit polyclonal anti-N antibody was validated in doi:10.1099/vir.0.79830-0<br>Mouse monoclonal anti-alpha-tubulin antibody. Validation statement from the company's website: Anti-a-Tubulin antibody, Mouse monoclonal (mouse IgG1 isotype) is derived from the hybridoma DM1A produced by the fusion of mouse myeloma cells (NS1) and splenocytes from BALB/c mice immunized with purified chick brain tubulin. The isotype is determined by a double diffusion immunoassay using Mouse Monoclonal Antibody Isotyping Reagents, Product Number ISO2.<br>Anti-rabbit secondary antibody, peroxidase-labeled. Validation statement from the company's website: affinity purified polyclonal antibody to rabbit IgG, both heavy and light chains (whole IgG), made in goat and labeled with horseradish peroxidase. Product is in lyophilized form. Each lot is tested to assure specificity and lot-to-lot consistency using an in-house ELISA assay.<br>Anti-mouse secondary antibody, peroxidase-labeled. Validation statement from the company's website: affinity purified polyclonal antibody to mouse IgG, both heavy and light chains (whole IgG), made in goat and labeled with horseradish peroxidase. Product has been cross-adsorbed to human serum to minimize cross-reactivity to human immunoglobulin. Product is in liquid form. Each lot is tested to assure specificity and lot-to-lot consistency using an in-house ELISA assay.

Eukaryotic cell lines

Policy information about cell lines and Sex and Gender in Research

Cell line source(s)	High Five cells (ThermoFisher Scientific, catalog number: B85502).<br>BHK-21 cells (clone BSRT7/5), constitutively expressing the T7 RNA polymerase. Reference doi:10.1128/JVI.73.1.251-259.1999 (1999). Cells were obtained from the Conzelmann group.
Authentication	Authentication was not done for this study.
Mycoplasma contamination	Mycoplasma test revealed no contamination.
Commonly misidentified lines (See ICLAC register)	No commonly misidentified lines were used in this study.
