# Supplementary figures and images for: Structural landscape of the respiratory syncytial virus nucleocapsids
Source: Nat Commun. 2023 Sep 15;14:5732. doi: 10.1038/s41467-023-41439-8 (PMC10504348; doi:10.1038/s41467-023-41439-8)

Supernatant after  
concentration

kDa

250

130

100

70

55

35

25

15

10

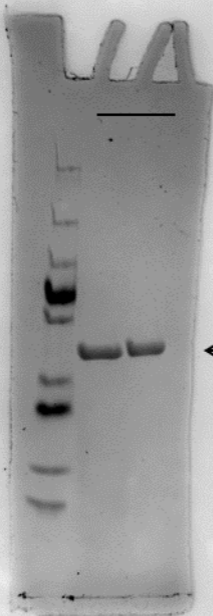

← N

Supplement: Supplementary file 6 — Source data [file 41467_2023_41439_MOESM6_ESM.zip › 41467_2023_41439_NOESM6_ESM/source/source-data-figure-9.pdf]
